# Utilizing confocal laser endomicroscopy for evaluating the adequacy of laparoscopic liver ablation

**DOI:** 10.1002/lsm.22464

**Published:** 2015-12-31

**Authors:** Crispin Schneider, Sean P. Johnson, Simon Walker‐Samuel, Kurinchi Gurusamy, Matthew J. Clarkson, Stephen Thompson, Yi Song, Johannes Totz, Richard J. Cook, Adrien E. Desjardins, David J. Hawkes, Brian R. Davidson

**Affiliations:** ^1^ Division of Surgery and Interventional ScienceRoyal Free CampusUniversity College LondonPond Street NW3 2QGLondonUK; ^2^ Centre for Advanced Biomedical ImagingDivision of MedicineUniversity College LondonLondonUnited Kingdom; ^3^ Division of Tissue Engineering and BiophotonicsKings College London Dental InstituteLondonUnited Kingdom; ^4^ Department of Medical Physics and BioengineeringUniversity College LondonLondonUnited Kingdom; ^5^ Centre for Medical Image ComputingUniversity College LondonLondonUnited Kingdom

**Keywords:** confocal laser endomicroscopy, liver ablation imaging, liver cancer, virtual histology

## Abstract

**Background:**

Laparoscopic liver ablation therapy can be used for the treatment of primary and secondary liver malignancy. The increased incidence of cancer recurrence associated with this approach, has been attributed to the inability of monitoring the extent of ablated liver tissue.

**Methods:**

The feasibility of assessing liver ablation with probe‐based confocal laser endomicroscopy (CLE) was studied in a porcine model of laparoscopic microwave liver ablation. Following the intravenous injection of the fluorophores fluorescein and indocyanine green, CLE images were recorded at 488 nm and 660 nm wavelength and compared to liver histology. Statistical analysis was performed to assess if fluorescence intensity change can predict the presence of ablated liver tissue.

**Results:**

CLE imaging of fluorescein at 488 nm provided good visualization of the hepatic microvasculature; whereas, CLE imaging of indocyanine green at 660 nm enabled detailed visualization of hepatic sinusoid architecture and interlobular septations. Fluorescence intensity as measured in relative fluorescence units was found to be 75–100% lower in ablated compared to healthy liver regions. General linear mixed modeling and ROC analysis found the decrease in fluorescence to be statistically significant.

**Conclusion:**

Laparoscopic, dual wavelength CLE imaging using two different fluorophores enables clinically useful visualization of multiple liver tissue compartments, in greater detail than is possible at a single wavelength. CLE imaging may provide valuable intraoperative information on the extent of laparoscopic liver ablation. Lasers Surg. Med. 48:299–310, 2016. © 2015 The Authors. *Lasers in Surgery and Medicine* Published by Wiley Periodicals, Inc.

## INTRODUCTION

Thermal ablation is a minimal invasive therapy, which can be used for the treatment of primary or secondary liver malignancy 1, 2. During the procedure, an ablation probe is inserted into the liver lesion where it causes thermal‐mediated denaturization of proteins which results in the destruction of cancer cells. Most commonly, heat is created by radiofrequency or microwave energy, but laser or high intensity focused ultrasound‐induced heat can also be employed 2. The ablation probe can be applied by percutaneous, laparoscopic, or open approaches. The laparoscopic approach allows easy access to tumors which are difficult to treat percutaneously such as those superficially in the liver or high under the diaphragm. It also permits use of laparoscopic ultrasound to localize the lesion and assess its relationship to local vital structures, hence reducing the incidence of collateral injuries 3. With small solitary liver cancers thermal ablation may produce comparable results to liver resection with a reduced morbidity and mortality 4. The main concern regarding thermal ablation is the high incidence of local recurrent disease and the main aim of ablation therapy is, therefore, to destroy not only the cancer harboring tissue but also a 5–10 mm safety margin to include any local satellite lesions. Ablated liver tissue changes in color from brown to white (total coagulation effect) but the spatial dimension of cellular destruction and functional damage extends well beyond this phenomenon, therefore making ablation monitoring crucial and mandating development of a visual inspection or detection device to locate the real damage margins. Using ultrasound for monitoring is unreliable because ablation therapy generates gas bubbles within the hepatic tissue which subsequently creates an acoustic shadow effect, negating any diagnostic value to the images 5, 6. Conventional computer tomography (CT) and magnetic resonance imaging (MRI) are commonly used for the postoperative assessment of ablation efficacy 7, 8 only, whereas intraoperative cone beam CT and open MRI scanners may be used but technical restrictions such as incompatibility with metal instruments (MRI) 9 and limited imaging quality (cone beam CT) make them impractical for the intraoperative evaluation of ablation therapy.

During thermal ablation, there is a significant change in tissue temperature which in itself can be regarded as a surrogate marker for cellular destruction 6. This relationship has been exploited for the purpose of noninvasive ablation monitoring, with MR‐ or CT thermography 9. In a more direct approach, tissue temperature can also be measured invasively by placing optical fibers under image guidance within liver tissue 5. This approach however confers the risk of needle tract seeding 10, that is, the risk of displacing tumor cells with the needle, into normal tissue and, therefore, disseminating the malignancy unnecessarily. Measuring changes in tissue elasticity by shear wave elastography has also been investigated for its utility in ablation monitoring 9, 11. The modalities listed above measure surrogate markers for tissue necrosis but a method of directly imaging the changes in the ablation zone has yet to be identified.

Confocal laser endomicroscopy (CLE) is a novel optical imaging technology that enables accurate microscopic assessment of *in vivo* histopathology. Its utilization in a variety of experimental and clinical scenarios has been increasingly reported. CLE utilizes a fiber optic element as its objective lens, allowing confocal microscopy with a micron‐scale resolution that enables visualization of subcellular details using endogenous or exogenous fluorescence. Exogenous fluorescence is provided by fluorescent dyes, which can be applied in a systemic (e.g., intravenous) or topical fashion and are commonly referred to as fluorophores. They enhance CLE image contrast by absorbing and emitting light at wavelengths that are specific for each individual agent and circumstance 12, 13.

CLE during laparoscopy or NOTES (Natural Orifice Transluminal Endoscopic Surgery) has been performed either with a rigid endomicroscope which contains the scanning optics in its tip 13 or with a slim, flexible optical fiber bundle which transmits light signals to laser scanning equipment situated at the far (e.g., extracorporeal) end of the intrusive fiber 14, 15. An example for a fiber‐based CLE system is Cellvizio™ (Mauna Kea Technologies, Paris, France), which is CE‐marked for clinical use in luminal endoscopy and can be operated with probes that have a diameter range of 0.3–4.5 mm. During endoscopy, Cellvizio™ can reveal histopathological cellular changes and has been shown to enhance accuracy in the diagnosis of malignancy and dysplasia 13, 16.

Liver ablation has been visualized using a bench‐top confocal laser microscope in a small animal model 17, but the diagnostic ability of a clinically approved CLE system to distinguish between healthy and necrotic liver tissue in a laparoscopic setting have yet to be tested. In this study, we aimed to evaluate if CLE imaging employing the Cellvizio™ system's potential can diagnose tissue necrosis in an *in vivo* porcine model of laparoscopic microwave liver ablation.

For establishment of the model, percutaneous microwave ablation was conducted under direct laparoscopic visualization. As the CLE system used in this study has a limited imaging depth of <100 μm, only superficial ablation zones were created and assessed. Optical scattering in hepatic tissue is very high and imaging depths are likely to be no better in any other optical technique. Imaging of subsurface ablation zones was not attempted. However, imaging of deeper ablated areas is of clinical relevance, and CLE via a placed optical fiber within the periphery of an ablation volume may be a potential approach to achieving this.

Previous groups evaluating laparoscopic CLE have used single fluorophores that are usually visible on CLE imaging within the blue‐light or near‐infrared spectrum 14, 18, 19. For the experiments presented here, dual wavelength CLE imaging at 488 nm (blue) and 660 nm (red) wavelength was facilitated by the fluorophores fluorescein and indocyanine green (ICG). By evaluating liver tissue at two discreet wavelengths and dissimilarly partitioned fluorophores, we aimed to maximize imaging information and highlight any potential advantages or disadvantages between the wavelengths and their corresponding fluorophores. The image acquisition software (ImageCell™) supplied with Cellvizio™ can compute basic image values (mean, median, maximum, minimum intensities). As well as comparing images, basic image summary statistics have been analyzed to establish if they can aid in defining the zone of tissue necrosis associated with microwave ablation.

This article makes the following contributions:

1. Description of normal porcine liver histology on CLE examination using fluorescein at 488 nm and indocyanine green at 660 nm illumination.
2. Evaluate the technical feasibility and limitations of CLE in an intraoperative setting.
3. Assess the ability of CLE to predict liver tissue necrosis induced by ablation therapy.

## METHODS

The experiments outlined below were performed in parallel with a different study conducted on the same animal, which has already been reported elsewhere 20. In brief; stereoscopic liver surface reconstruction and optical tracking were used to develop an image guidance system for laparoscopic liver surgery.

### Sequence of Events

The experimental flow model is listed below, sequentially:

1. Laparoscopic microwave ablation.
2. Recovery period of 7–10 days.
3. Laparoscopic CLE image acquisition (following laparoscopic liver resection in two cases).
4. Post mortem liver biopsies.

### Study Animals

Following approval by the local animal ethics committee, experiments were performed at Northwick Park Institute for Medical Research (London). A total of four female landrace mini‐pigs with a bodyweight ranging between 50 kg and 80 kg were studied. Each animal underwent two procedures under general anesthesia on separate days with a 7–10 day recovery period in between. On the first procedure, laparoscopic liver ablation zones were produced (Medical microwave systems research group, Bangor University, UK). During the second procedure CLE images and liver biopsies were obtained. In two animals a left laparoscopic hemihepatectomy was carried out prior to imaging, to evaluate CLE examination of the surgical resection margin.

### Preparation and Anesthesia

Following best practice animal husbandry, the mini‐pigs were acclimatized to their surroundings with free access to food and water at least 1 week prior to experiments. To avoid intestinal distension animals were fasted 24 hours prior to surgery but had continual access to water. Oral endotracheal intubation facilitated general anesthesia with 2% Isoflurane in oxygen in a recovery (ablation) or nonrecovery (CLE imaging) algorithm with 200 mg Pentobarbitone euthanasia at termination of surgery. Orogastric intubation decompressed the stomach during laparoscopy. All procedures were carried out with the animals in a supine position.

### In Vivo Image Acquisition

Following establishment of the pneumoperitoneum, abdominal contents were visually inspected for potential ablation induced collateral injuries. All imaging experiments were recorded with the laparoscopic camera and stored as digital files. Position of the Cellvizio™ probe tip in relation to the ablation zones examined could be identified on video and correlated with the recorded CLE images for future analysis. Immediately before liver imaging, a standardized Cellvizio™ system calibration procedure 21 was carried out in theater. Baseline CLE images were recorded over normal and ablated liver, respectively. Fluorescein sodium (molecular weight 376.27 g/mol—henceforth called “fluorescein”) and ICG (molecular weight 774.96 g/mol) were mixed with 50 ml 0.9% sodium chloride or 5% dextrose, respectively, just prior to intravenous injection. Intravenous injection of fluorescein (at 4–7 mg/kg) and indocyanine green (at 0.3–0.5 mg/kg) was carried out sequentially with an interval of 30–60 minutes between injections. Fluorescein and ICG acted as fluorophores for CLE imaging at 488 nm and 660 nm wavelength, respectively. CLE image acquisition was carried out with separate systems for each individual wavelength (Cellvizio™ 488 nm and Cellvizio™ 660 nm, Mauna Kea Technologies, Paris). In addition to this protocol, two animals underwent fluorophore injection 15 minutes prior to ablation therapy to determine if the time point of injection impacted on CLE imaging characteristics. In preliminary bench experiments, it was confirmed that the illumination—emission spectra were so widely separated, that there was no cross‐talk between the 488‐fluorescein and the 660‐ICG systems. An initial image sequence was recorded to monitor and confirm distribution of the fluorophores in the liver. This was followed by several short sequence acquisitions taken in equal numbers from normal parenchyma and ablated regions. Short sequences ranged from 10 to 20 seconds and were later analyzed to determine variations of relative fluorescence between healthy and ablated liver tissue.

When visualization became impeded by blood or other detritus that had attached itself to the tip of the probe, it was cleaned with normal saline. This reflects the value of an imaging scheme rather than a single fiber spectrometer. In the latter case a low signal could represent either a lack of fluorophore or a fouled fiber tip—enhancing the risk of a false reading from a given sampling site.

Before experiments commenced, a training session was conducted to familiarize surgeons with CLE probe handling during laparoscopy. Particular attention was paid to probe tip pressure because too little pressure resulted in insufficient tissue contact, whereas too much pressure severely diminished fluorescence intensity. Both extremes resulted in the loss of any measurable fluorescence intensity and hence a black image which again demonstrates the value of an imaging system rather than a spectrometer fiber. The training session allowed surgeons to find a narrow range of tip pressure that enabled image acquisition at a visibly stable fluorescence level. From a surgical technique point of view, deliberate adjustment of pressure within this narrow window was very challenging, without haptic feedback so no formal quantification of probe tip pressure was undertaken. None of the image data acquired during the training session was used for analysis.

### Operative Technique

Pneumoperitoneum was established using either the Veress technique or port insertion under direct vision. A periumbilical 10 mm port was used for the laparoscopic camera with additional 5 mm and 10 mm ports inserted in the right and left upper quadrants. For ablation, one additional 5 mm port was inserted in the right subcostal region. Liver resection was performed using four ports in addition to the camera port; three in the right upper and one in the left upper abdomen. Pneumoperitoneum was constantly monitored and maintained at 8–12 mm Hg. The porcine equivalent of a left hemihepatectomy was carried out without vascular inflow occlusion. A portable ultrasonic scalpel (Sonicision™, Covidien, Dublin, Ireland) was used for transection of the liver parenchyma. Small vessels were controlled with ultrasonic scalpel or metal clips, whereas major vessels were divided with a laparoscopic stapling device (Echelon Endopath™, Ethicon Endo‐Surgery, Blue Ash).

### CLE Equipment

Experiments were performed sequentially in a “dual wave length approach” using both 488 nm (blue) and 660 nm (red) laser lines which were connected to separate CLE imaging systems (Cellvizio™ 488 nm and Cellvizio™ 660 nm, Mauna Kea Technologies, Paris). For CLE image acquisition 0.5–1.5 mm, flexible confocal probes (MiniZ, Ultra MiniO and S1500, Mauna Kea Technologies, Paris, France) were used, all compatible with conventional endoscopic instrument channels. The CLE imaging systems control laser illumination and fiber scanning, delivering 12 frames per second which enables near real‐time image acquisition. Images at different wavelengths were recorded “in turn,” meaning without parallel coregistration of the red‐ and blue‐light laser systems. Imaging parameters were probe dependent with 1.4–3.5 μm lateral resolution, 10–30 μm axial resolution, 0–70 μm imaging depth and a 240–600 μm^2^ fields of view.

**Figure 1:**
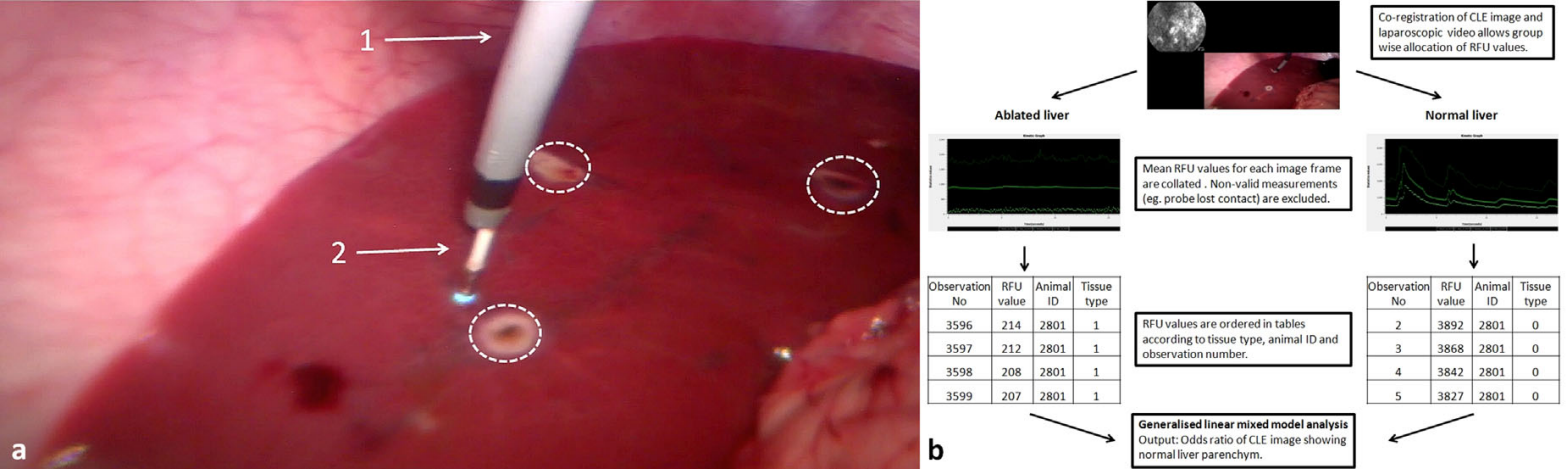
(**a**) Laparoscopic CLE imaging of porcine liver with fluorescein/488 nm. The ablated regions are highlighted with dashed circles. (i) Steerable catheter inserted through a laparoscopic port; (ii) CLE probe with blue light reflex at the tip. (**b**) Workflow of statistical analysis.

The confocal probes were inserted into the peritoneal cavity through the lumen of a steerable catheter (Agilis™ St. Jude Medical, Saint Paul, MN) which was itself introduced through a 5 mm laparoscopic port. The steerable catheter, designed for intravascular use, allowed subtle manipulation of the probe tip during laparoscopy (Fig. 1a). A technical note on this approach to probe manipulation has been reported elsewhere 22.

### Preparation of Tissue Samples

Following termination of the animals, the ablation zones and the surrounding healthy liver were resected for histological analysis. During surgery, if more than one ablation zone was examined, they were assigned numbers and their anatomical position was recorded during laparoscopy with the intention to facilitate postoperative CLE image analysis for each individual lesion. No attempt was made to perform a more detailed spatial correlation between CLE‐ and histopathological images because the CLE probe's field of view (<300 μm^2^) was too small to accurately localize with markings that would have been visible on laparoscopy.

Samples were fixed in formalin and then embedded in paraffin. Tissue samples were sliced with a histiotome in a direction parallel to the liver surface. These slices were placed on glass slips and stained according to a standard Haematoxylin & Eosin protocol.

### Statistical Analysis

Using an integrated software package (ImageCell™, Mauna Kea Technologies, Paris, France) mean fluorescence values for each image frame were recorded and exported for statistical comparison. The values were termed “relative fluorescence units” (RFU).

Negative RFU values can potentially represent either image noise, or loss of autofluorescence due to prolonged illumination causing a “photo‐bleach” effect 21. To avoid misinterpretation, negative RFU values were therefore thresholded at zero. Image sequences for each separate animal/imaging session were divided into two groups: normal liver parenchyma and ablated liver parenchyma, depending on the type of liver tissue they were recorded from. The mean RFU value for each image frame was compared between these two groups. Because of its technical design, each recalibration of the Cellvizio™ system influences the RFU value. Data was, therefore, compared only for the same calibration event and experiment and normal and ablated tissues were only compared for the same animal. To adjust the data for erratic probe movement all laparoscopic videos were reviewed. If there was a clear disconnection between the probe tip and the liver surface the relevant data sequence was excluded from analysis.

Evaluation of data distribution using the Kolmogorov–Smirnov test showed that the values in both groups were nonparametric. There was an unequal number of frame values in both groups, which would invalidate routine paired group comparison (e.g., paired Wilcoxon rank sum test). For this reason data were analyzed employing a generalized linear mixed model (GLIMMIX) which allows for the incorporation of fixed (RFU, ablated vs. normal liver) and random (animal ID) effects. This facilitated comparison of unequally sized groups and accommodated changes in the RFU values with each new calibration of the Cellvizio™.

The GLIMMIX analysis was set up to model the probability of the probe being on normal (i.e., nonablated) liver parenchyma. The measured RFU value was set up as fixed effect and the animal ID (and therefore potential changes with each new calibration) was set as a random effect in the model. To reduce data skew and kurtosis, mean values for each frame were transformed using the root‐square transformation. The GLIMMIX analysis was based on the estimation of residual marginal expansion pseudo‐likelihood. Glimmix result output is given as the change in odds ratio for an increase in RFU at a standardized value. For example, an odds ratio of 2.0 at standardized value of 100 to 101 RFU means that the odds ratio of liver parenchyma being normal increases by two if the RFU value increase by one from 100 to 101.

To test the validity of the GLIMMIX model a receiver operating characteristic (ROC) analysis was performed. The covariates and intercepts provided by the GLIMMIX analysis were used to calculate the log odds, odds and finally the probability of identifying nonablated tissue. Each single probability value corresponded to a single observation (mean RFU per frame), a specific animal ID and a tissue type, which enabled us to perform a ROC analysis based on this data. Because animal ID was a random effect, the same RFU value would result in different probability values for the individual animals. This relationship explains why based on our data, no single RFU cut‐off value can be estimated as an optimal threshold to distinguish ablated from nonablated tissue.

Statistically significant differences were accepted if *P *< 0.05. Statistical analysis was performed using SPSS™ Version 21 (IBM, Armonk, NY) with exception of the GLIMMIX analysis which was carried out with SAS™ Version 9.4 (SAS Institute, Cary, NC). Nontransformed group values are given as median and interquartile range. The workflow for the statistical analysis is shown in Figure 1b.

## RESULTS

### Descriptive Evaluation of Liver Histology

#### General histology

The histological samples obtained within 7–10 days of microwave ablation showed different degrees of cellular injury in the ablated areas. Centrally, within the ablation zone, tissue appeared macroscopically white and on histology, showed complete coagulation necrosis. Surrounding these areas was a rim of tissue that showed signs of sinusoidal obstruction, cell shrinkage and disruption of lobular architecture. On macroscopic inspection these areas were difficult to distinguish from the normal liver tissue in the periphery, validating the use of a sensitive imaging instrument for this purpose.

#### CLE evaluation of fluorescein at 488 nm—normal liver

CLE images are shown adjacent to histology sections, obtained from normal porcine liver after conclusion of the experiments. To facilitate comparison, CLE images are shown in parallel to histology slides with comparable architectural features from the same animal.

**Figure 2:**
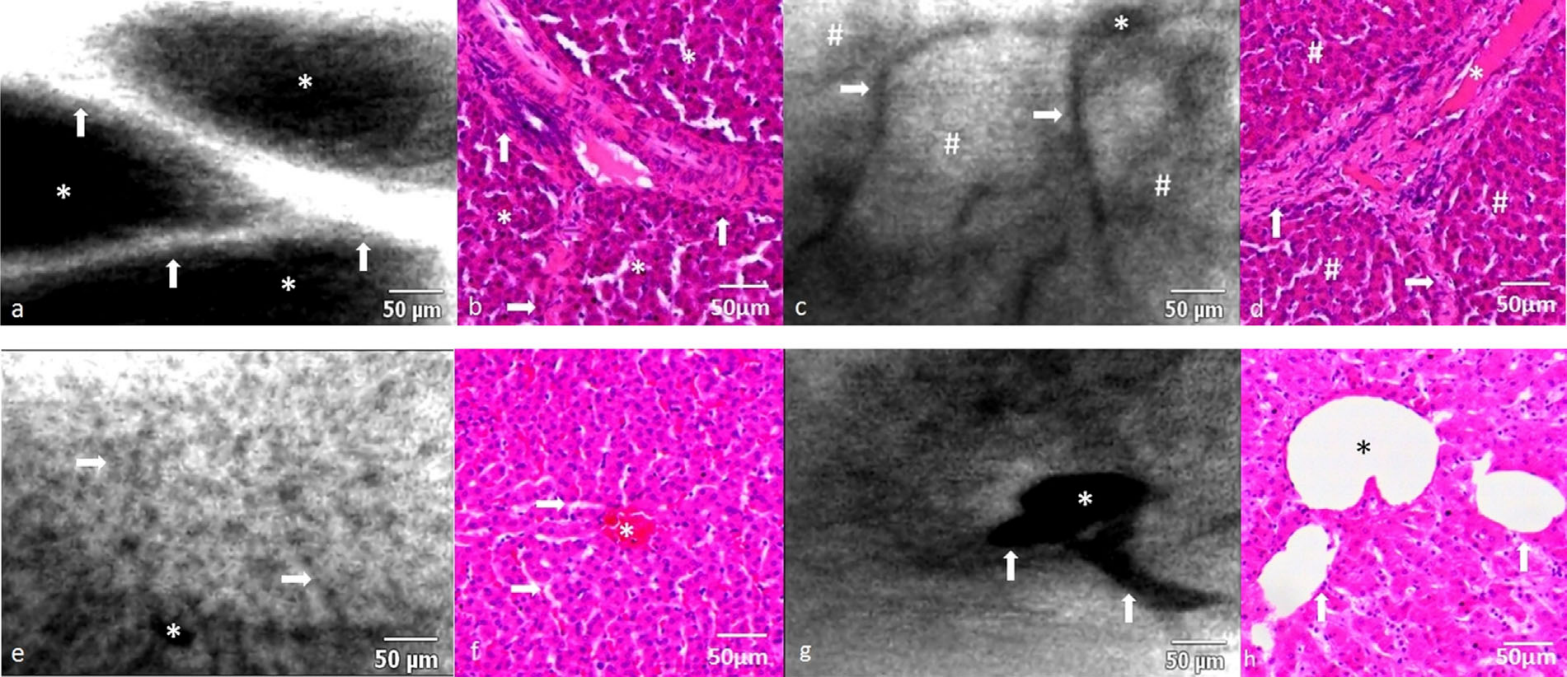
Comparison of CLE imaging with fluorescein/488 nm and H&E liver histology. (**a** and **b**) Strong fluorescence signal in the intravascular compartment during the inflow phase. *Liver lobules; arrows, interlobular vessels. (**c** and **d**) Shift of fluorescence signal from the intravascular‐ to the intracellular compartment in the parenchymal phase. ^#^Liver lobules; *vessel bifurcation; arrows, vessel branches. (**e** and **f**) Pattern of sinusoidal architecture. *Horizontal sinusoid; arrows, perpendicular sinusoid. (**g** and **h**) Vessel bifurcation of a centrilobular vein. *Vessel lumen; arrows, vessel branches.

When using 488 nm CLE, no auto‐fluorescence signals were recordable from the tissue before fluorescein administration. Intravenous fluorophore injection was followed by an “inflow phase” where the fluorophore is distributed and taken up by the liver parenchyma. This phase lasts for approximately 7–10 minutes after injection and is characterized by bright signals in blood vessels contrasting with dark lobule areas (Fig. 2a and b). Using CLE imaging of fluorescein at 488 nm, individual erythrocytes flowing through blood vessels could be observed with greater regularity than was possible with CLE imaging of ICG at 660 nm. Unfortunately, the limited image resolution made a more detailed analysis of blood vessel flow difficult at either wavelength.

The inflow phase was followed by the “parenchymal phase” when the fluorescence pattern is reversed as the fluorophore has been redistributed from the vasculature into the liver parenchyma. During this phase, the vasculature is devoid of fluorescence signal and can be visualized as dark lines of increased contrast (i.e., absence of fluorescence) that surrounds the liver lobules, which had by then become rich in fluorescein (Fig. 2c and d). CLE imaging of fluorescein at 488 nm allowed sinusoid structure and individual hepatocyte cords visualization (Fig. 2e and f). Usually, a distinction between different types of vessels (arterial/venous) is not feasible, but in some instances the location and morphology of blood vessels can reveal its specific characteristics (Fig. 2g and h).

#### CLE of ICG at 660 nm—normal liver

Similar to the fluorescein system, 660 nm CLE illumination of ICG, demonstrated no tissue auto‐fluorescence and thus no imaging of liver architecture was possible prior to fluorophore administration.

**Figure 3:**
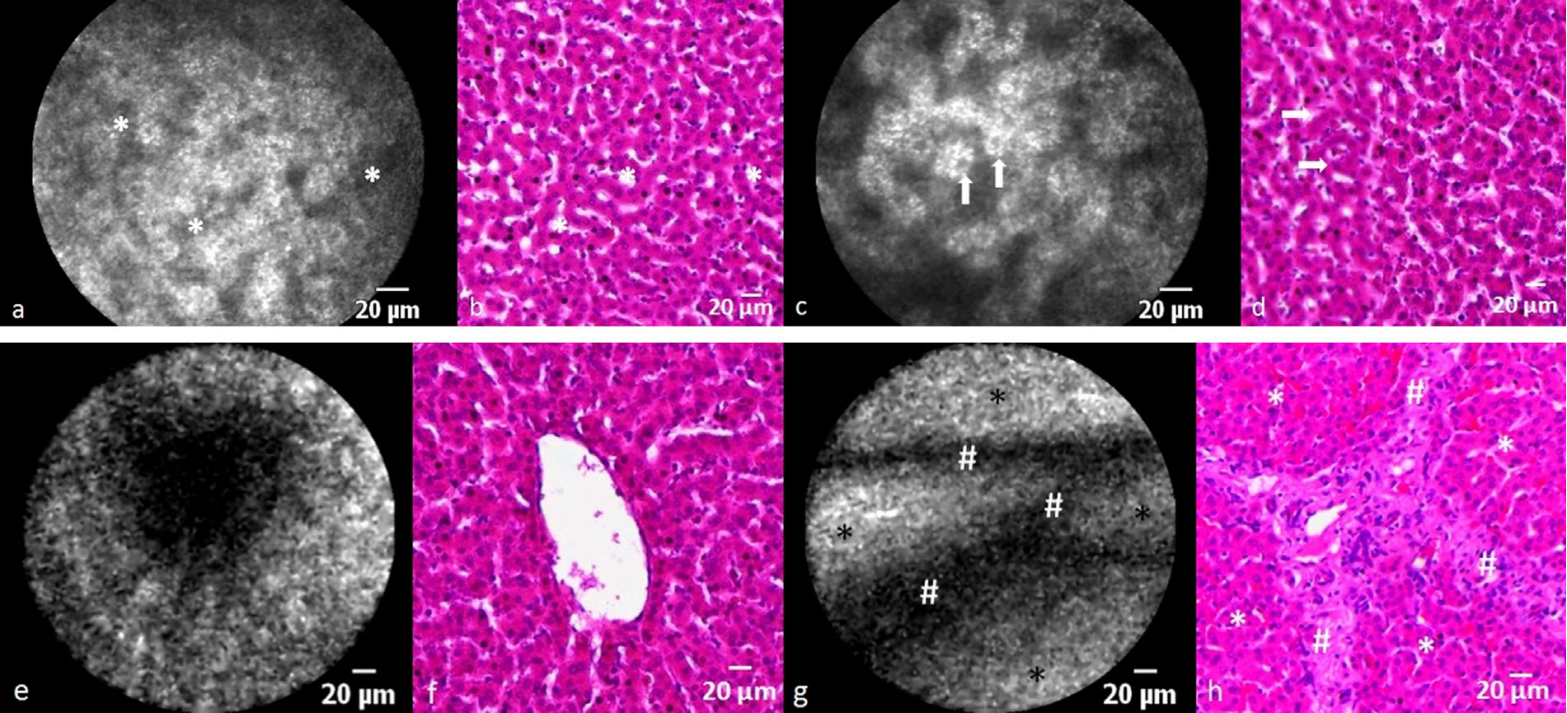
Comparison of CLE imaging with ICG/660 nm and H&E liver histology. (**a** and **b**) Typical sinusoidal architecture of normal porcine liver. *Sinusoids. (**c** and **d**) Hepatocyte nuclei appear as intracellular contrast sparing. arrows, nuclei. (**e** and **f**) The dark central area represents a centrilobular vein. Note how the vessel lumen appears ragged because ICG does not accumulate in the vascular endothelial cells. (**g** and **h**) Interlobular septations appear as linear contrast sparing areas between liver lobules. #Septation; *liver lobule.

**Figure 4:**
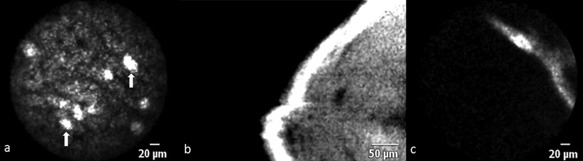
(**a**) Bright foci of fluorescence (arrows) at the later stages of CLE imaging with ICG/660 nm may correlate with areas where bile juice accumulates. The surface of the resected liver is mostly devoid of fluorescence but occasionally band‐like fluorescence signals can be seen at 488 nm (**b**) and 660 nm (**c**) wavelength.

In contrast to CLE of fluorescein under 488 nm excitation, it was not possible to visualize any significant fluorescence within the intravascular compartment during the “inflow phase.” The combination of ICG and 660 nm CLE, however, appeared to be better suited to imaging the sinusoid structure within liver lobules (Fig. 3a and b). Because ICG is rapidly taken up and cleared by the hepatocyte cytoplasm, the hepatocyte nuclei were seen outlined as dark spots within the liver cells (Fig. 3c and d). Central lobular veins that drain the blood toward the hepatic venous system could be seen as round dark structures within the center of a lobule (Fig. 3e and f). Because the vasculature and connective tissues were both devoid of fluorescence signal in the parenchymal phase, blood vessels could not be distinguished from interlobular septations when using ICG as sole fluorophore (Fig. 3g and h). Within this limitation, the visualization of fibrous tissue septations between lobules was readily reproducible with ICG CLE at this wavelength. Between 30 and 40 minutes after ICG infusion, bright spots were seen appearing throughout the liver parenchyma (Fig. 4a). This phenomenon possibly represents areas of ICG accumulation (e.g., bile juice).

#### Probe manipulation, placement, and image quality

As the CLE probe has no inherent navigability, the use of an intravascular catheter (Agilis™ St. Jude Medical, Saint Paul, MN) afforded free rotation, flexion, and retroflexion to 180°. Familiarity with the steering system allowed instant successful use of the CLE instrument without additional prior expertise or training.

This degree of maneuverability allowed access to virtually any area on the liver surface (Fig. 1a). The main detractor to image quality was the respiratory movement of the liver. This made methodical and complete examination of liver tissue difficult because tissue contact loss would frequently occur with each respiratory cycle. The problem of respiratory interference to CLE signal was greater on the anterior and superior surfaces of the liver and was compounded by pooling of fluids or blood on the liver surface which increased probe tip slipping. The size of the steerable access catheter allowed parallel insertion of two CLE probes, this method however made overall handling difficult and impaired image quality.

#### Evaluating ablation zones and liver resection surface

**Figure 5:**
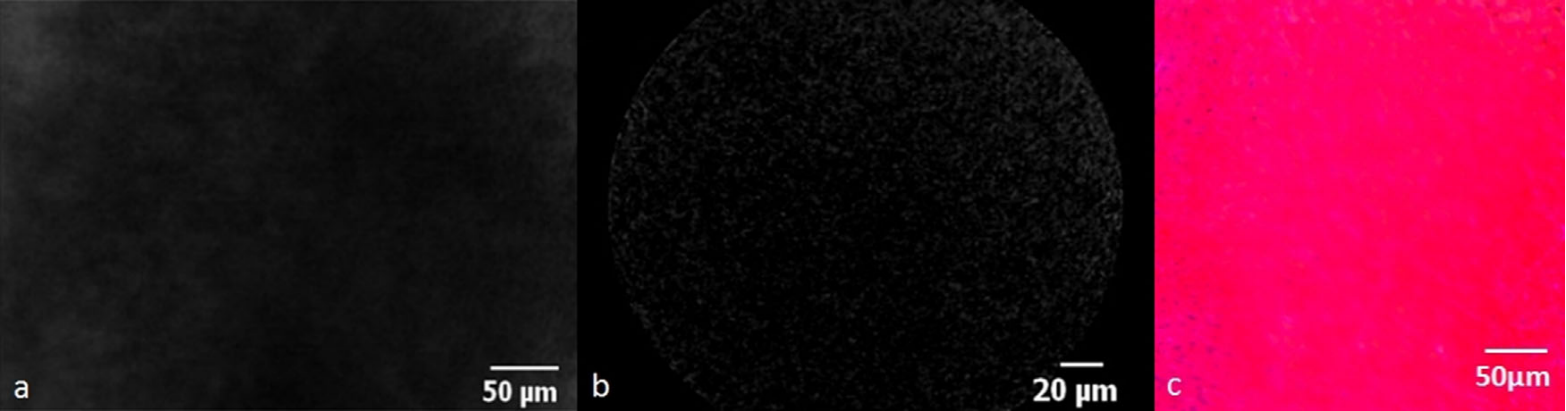
Areas of complete necrosis with fluorescein/488 nm (**a**) and ICG/660 nm (**b**) show loss of fluorescence intensity which correlates with complete destruction of hepatic architecture on H&E histological analysis (**c**).

**Figure 6:**
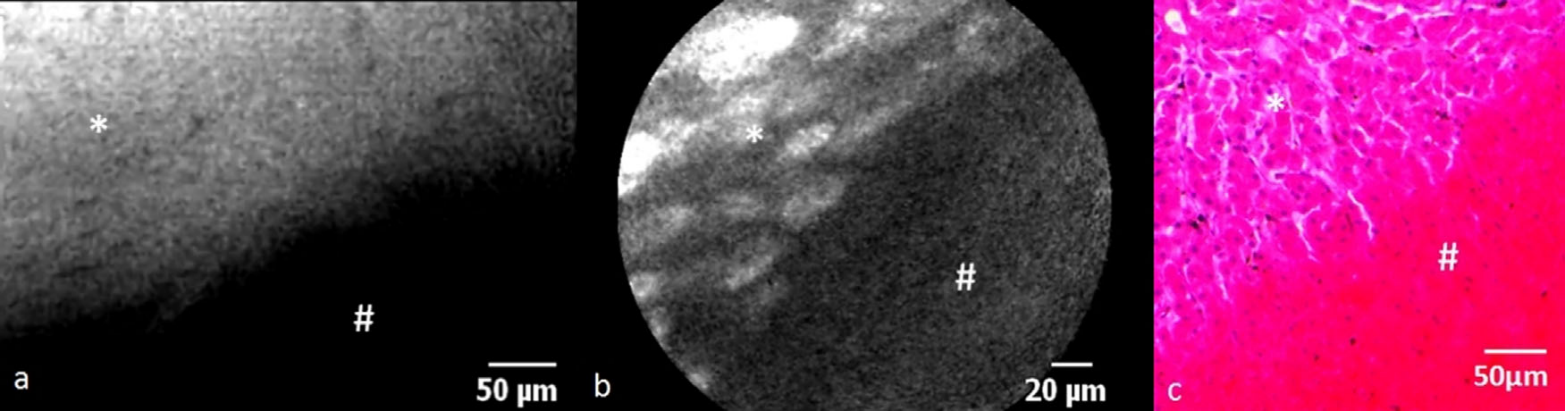
The border between complete necrosis (#) and partial cellular injury (*) can be visualized with fluorescein/488 nm (**a**) and ICG/660 nm (**b**). The corresponding H&E histological appearance can be seen in (**c**).

In ablated liver tissue, the characteristics of CLE images recorded with fluorescein and ICG were similar in all the animals studied. Whether the fluorophore was injected before or after ablation was carried out, did not affect visualization of tissue characteristics. In the majority of the ablation zone, no fluorescence was detectable which corresponded to zones of ablation induced injury on histology (Fig. 5). When examining the border region between normal and ablated liver, the change from high to low fluorescence zone could often be observed in the same field of view (Fig. 6). Because it was technically difficult to stabilize the probe over a very small area (<1 mm), no differentiation between zones of cellular apoptosis and necrosis could be made. Similar to zones of ablated liver, the liver resection surface (divided using the ultrasonic scalpel) did not show any significant fluorescence signals. If signals were recorded on the resection surface, they usually originated from linear structures of high signal intensity that potentially represent vessels (Fig. 4b and c) or zones of fluorophore extravasation following the destruction of fluorophore containing vessels.

#### Analysis of fluorescence values

**Figure 7:**
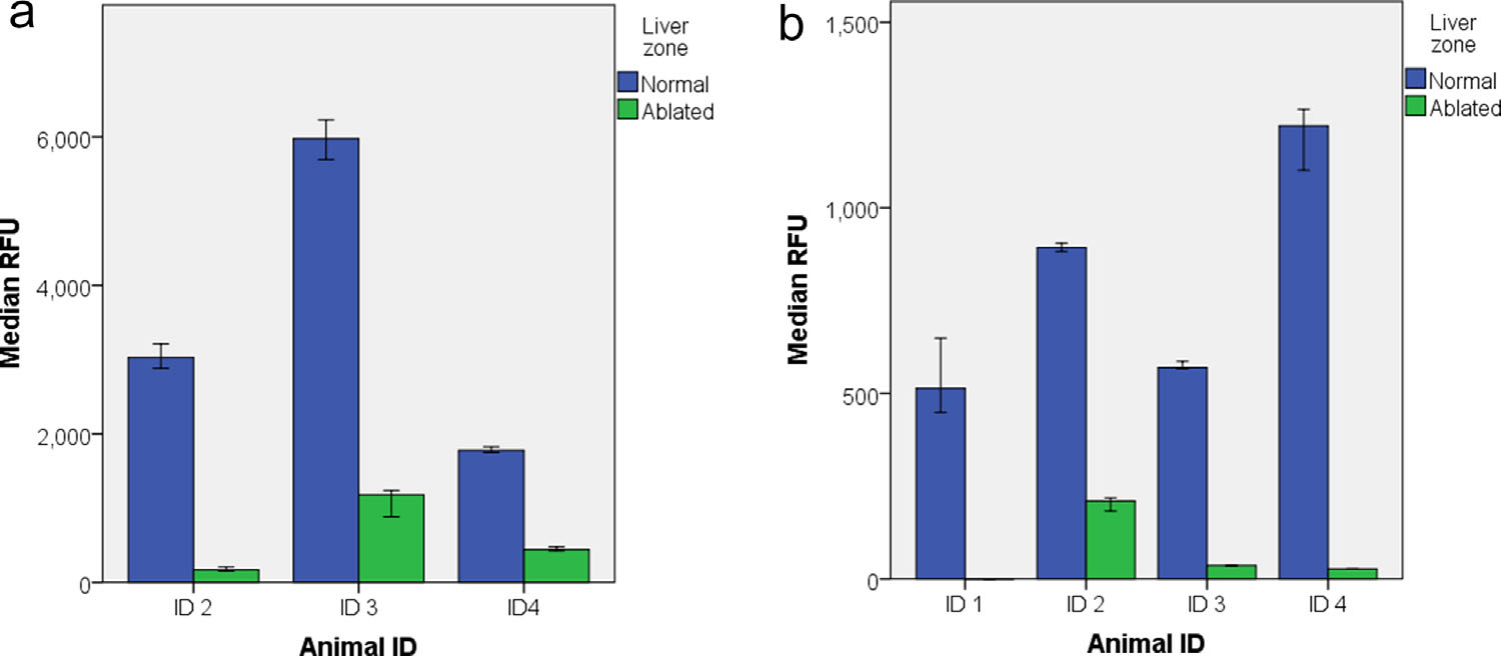
(**a**) Median RFU values ± 95%CI for each animal liver studied with fluorescein/488 nm. (**b**) Median RFU values ± 95%CI for each animal liver studied with ICG/660 nm. The bar for ablated tissue in animal ID 1 is not visible because the median and 95%CI is zero.

Overall RFU values measured in nonablated versus ablated tissue decreased by 75–94% and 77–100% for CLE imaging with the fluorescein/488 nm system and the ICG/660 nm system, respectively (Fig. 7). In Table 1, median RFU values for each animal and tissue type are shown with ablated tissue further subdivided into individual lesions.

**Table 1:** Median RFU Values for Nonablated and Ablated Tissue as Well as for Each Individual Lesion That Was Examined During Experiments

Animal ID.	Type of tissue	Fluorescein and 488 nm	ICG and 660 nm
1	Nonablated	*	514 (624)
	Ablated	*	0 (20)
	RFU change in %		100%
	Lesion 1	*	20 (18)
	Lesion 2	*	0 (0)
2	Nonablated	3,030 (1,868)	893 (240)
	Ablated (Lesion 1)	169 (862)	210 (123)
	RFU change in %	94%	77%
3	Nonablated	5,978 (3,782)	570 (254)
	Ablated	1,177 (949)	37 (29)
	RFU change in %	80%	94%
	Lesion 1	1,177 (949)	17 (8)
	Lesion 2	*	63 (106)
	Lesion 3	*	36 (2)
4	Nonablated	1,778 (846)	1,221 (532)
	Ablated	443 (627)	27 (3)
	RFU change in %	75%	98%
	Lesion 1	838 (871)	24 (3)
	Lesion 2	381 (192)	20 (16)
	Lesion 3	953 (707)	27 (1)

The interquartile range is given in brackets. RFU change states the decrease of median RFU values in nonablated (set as 100%) vs. ablated tissue. *No image acquisition for these lesion due to technical issues.

With CLE of fluorescein using 488 nm excitation, a standardized RFU value change from 41 to 42 (square root) increased the odds ratio of liver parenchyma being normal (i.e., nonablated) by 1.16 (*P* < 0.0001; CI 1.15–1.17). At this fluorophore and wavelength combination the variation introduced by imaging individual animals and recalibrating the Cellvizio™ system did not contribute to the fit of the model as a random effect with a covariance parameter of 1.66 ± 1.66 (SEM, *P *> 0.05). The covariance parameter estimation indicates to what degree the random variable affects the fit of the model (i.e., it aids in predicting outcome) of the model and its associated standard error gives a measure of repeat sampling variability. This means that the same RFU values could be applied across different animals in the study to predict the presence of healthy liver tissue. ROC analysis resulted in an area under the curve of 0.955 ± 0.003 (*P* < 0.0001, CI 0.95–0.96).

A standardized RFU value change from 17 to 18 (square root) with CLE imaging of ICG at 660 nm increased the odds ratio of liver parenchyma being normal by 1.58 (*P* < 0.0001, CI 1.54–1.63). Variation introduced by individual animals and recalibration did significantly contribute to the fit of the model as a random effect with a covariance parameter estimation of 4.6 ± 3.8 (SEM, *P* < 0.05). This means that the predictive RFU values were not interchangeable for different animals in the study as each individual's fluorophore distribution pattern within the areas of interest, were unique to that animal. ROC analysis resulted in an area under the curve of 0.999 ± 0 (*P* < 0.0001, CI 0.998–1.0).

## DISCUSSION

This study has demonstrated that confocal laser endomicroscopy can be used *in vivo* to distinguish thermally ablated liver, from healthy liver, during laparoscopic surgery. The main discriminating factor between healthy and ablated tissue was loss of fluorescence intensity as expressed by RFU. This phenomenon was independent from the time point of fluorophore injection, which means that it is unlikely to be caused by the destruction of fluorophore carrying vasculature. It is proposed that the destruction of fluorophore containing cellular and intercellular structures that occurs during liver ablation is the most likely explanation for this finding.

Evaluation of ablated liver tissue is of relevance because similar to surgical excision of malignancy, a complete ablation margin around a treated lesion is necessary to optimize patient survival 23. An important limitation of thermal ablation is that it is relatively contraindicated when the tumor is close to vulnerable structures such as major bile duct and blood vessel structures 24. Proximity to large blood vessels also renders ablation less effective and predictable because of the “heat sink effect” 24. Although not demonstrated here, CLE probes could be deployed close to vulnerable structures to detect thermal injury and allow the progress of ablation to be monitored. Because the approach evaluated in this article enables direct visual tissue assessment in small and distinct areas of liver parenchyma, it may be advantageous compared to measuring surrogate markers of tissue necrosis such as temperature.

An analysis based on generalized linear mixed modeling has indicated that loss of fluorescence intensity can be used *in vivo* to identify ablated or nonvital liver tissue. This type of analysis was chosen over and above a more simplistic group‐wise comparison because of the specific constraints that the collected data presented. Firstly, we had to account for comparing two different types of tissues in the same animal (normal and ablated); secondly, the tissue types were repeatedly measured to account for the inconsistency introduced by CLE probe movement and a small field of view; thirdly, an incongruent number of mean RFU values in each group resulted from having a different number of frames available for analysis. Lastly, the absolute values of the measured RFU were not comparable between different experimental days because each new calibration of the Cellvizio™ altered the RFU value.

The general linear mixed model analysis allows for all these considerations and takes into account the correlation in measurements between different tissues in the same animal. It is also able to handle nonparametrically distributed data. A separate ROC analysis has been used to confirm the validity of the model. Before fluorescence values can be used to inform clinical decision making, however, it is imperative that standardized, absolute fluorescence values are established. If an ablation dependent variability in absolute fluorescence can be validated in more extensive datasets, it has the potential to be transformed into a computational image recognition algorithm. This algorithm could then be integrated into a clinical imaging system with the ability to monitor and map liver ablation.

Each frame value was treated separately because unavoidable probe movement meant that some sections of tissue may just be represented by a single frame. Although a single image frame may not provide enough data for clinicians to evaluate tissue, it could provide sufficient data for an automatic tissue evaluation algorithm based on fluorescence intensity.

A description has been provided comparing key characteristics of liver tissue architecture as imaged on fluorescein and ICG facilitated CLE examination at 488 nm and 660 nm wavelength. To account for temporal variations in imaging characteristics following fluorophore injection, a categorization into hepatic “inflow” and “parenchymal” phases has been proposed here. The inflow phase lasts approximately 7–10 minutes and takes place while the fluorophore is mainly concentrated in the hepatic vasculature. This blends over into the parenchymal phase when the bulk of fluorophore concentration has been redistributed into the intracellular compartment. Although a number of articles have evaluated CLE of the liver for different indications in animals and humans, 14, 15, 19, 25 no report to date has made a comparative description by employing a dual wavelength approach. Because fluorescein and indocyanine green have different distribution properties in tissues 18, 26, 27, they could be employed sequentially and within a time frame to allow examination of a particular area of interest. Overview images obtained during the inflow phase had different characteristics for both fluorophore and wavelength combinations. When focusing on blood flow evaluation in this phase, CLE examination of fluorescein with the blue‐light system was advantageous because it enabled regular visualization of erythrocyte movement within sinusoids and larger parenchymal vessels. Although ICG is similarly distributed through the vasculature, it was not possible to detect intravascular ICG mediated fluorescence in this study. A group investigating near‐infrared CLE imaging of ICG in patients with liver disease were able to visualize blood flow in 8 out of 21 patients 18 possibly reflecting low/slow function of the hepatocytes in these particular eight cases. No ICG related fluorescence was visualized within sinusoidal vessels which was also the case in our experiments. It is not possible to draw any conclusions from this because the employed CLE systems have different specifications and with only four subjects in our study, the inability to visualize hepatic blood flow may have been accidental but it is tempting to ascribe it to good hepatocyte affinity for ICG in health, allowing a one‐pass clearance effect.

In the parenchymal phase, CLE of the fluorescein/488 nm system was better suited to imaging blood vessel morphology because low contrast areas correlated better with blood vessels, whereas these areas in the ICG/660 nm system were consistent with both fibrous tissue septations and vasculature. A more homogenous and intense distribution of fluorescence throughout the liver tissue indicated that fluorescein accumulated in hepatocytes and other cells (e.g., fibrocytes and vascular endothelial cells). In comparison, ICG CLE at 660 nm revealed a high affinity of ICG to hepatocytes with nuclear sparing, providing a strong contrast. Visualization of interlobular septation architecture appeared to be better suited to the 660 nm CLE system with ICG, which was able to clearly delineate liver lobules from surrounding fibrous tissue. At 30–40 minutes after ICG injection areas of 5–20 μm^2^ could be observed that accumulated high fluorescence intensity. Given that ICG is known to be rapidly excreted via bile 26, it is proposed that these signals may represent bile canaliculi, the smallest division of bile ducts . The fluorophore choice of fluorescein and ICG was guided by potential clinical applicability. There are many other potential fluorophores for 488–660 nm CLE, but none are currently approved for clinical use or have the same low risk profile as fluorescein and ICG 26, 27. The latter two fluorophores have been used in CLE studies of the human liver in the past 18, 19 which could aid in the transferability of our findings to a clinical evaluation of CLE imaging in liver ablation.

Overall, the detection sensitivity of ICG fluorescence intensity at 660 nm was lower when compared to fluorescein at 488 nm wavelength. This was likely due to the fact that the optical absorption maximum of ICG is 800 nm (near‐infrared spectrum) so our excitation efficiency was poorer at 660 nm, and while not optimal, was adequately within the excitation range to excite fluorescence emissions for this fluorophore. This illumination wavelength is a manufacturer preset and cannot be altered. Near‐infrared CLE imaging, however, is not available yet in a CE marked system and we felt that adequate signal was achieved in the system described, as the fluorophore has attractive properties relevant for clinical liver and bile duct imaging 26.

Although porcine models are frequently used to evaluate novel surgical technologies 28, 29, the resulting findings have to be interpreted with caution. Firstly, porcine liver histology differs from humans with more pronounced interlobular septations. Despite these differences, authors with extensive experience in CLE imaging of the liver state that data acquired from animal studies can be regarded as equivalent to human data 12. Secondly, we found during laparoscopy that the pig's distinctive multilobulated liver configuration altered the handling and manipulation of the liver considerably in comparison with the expected characteristics in humans. Although the number of animals tested was small, the extensive data acquisition was comparable to similar studies published by other groups 14, 30. The acquired number of images was sufficient to describe dual wavelength CLE imaging features and to evidence the feasibility of using fluorescence values to distinguish between vital and ablated liver parenchyma in an individual. Establishing large animal models of liver malignancy is expensive, time consuming, and not routinely employed for research purposes 31. Therefore, the pig model employed in this article solely focused on assessment of ablated tissue and did not account for the presence of malignant tissue with its commonly associated vascular disturbances, as would be encountered during clinical liver ablation procedures. The advantage of CLE imaging compared to a simpler fluorescence detection device is that it has the ability to demonstrate tissue architecture and thus discriminate between malignant and benign tissue 13, but due to the limitations of the porcine model this aspect could not be evaluated in this study. In addition, the image quality alludes to fouling of the tip—a danger in single fiber spectrophotometry systems reporting no signal due to absence of fluorophore or a fouled tip?

The relation and distribution of fluorescence characteristics in healthy and ablated liver tissue demonstrated in this article indicate that CLE may well have utility in ablation monitoring but further research is required. Before absolute RFU values can be compared between different experiments or between research groups, the Cellvizio™ platform or alternative CLE imaging systems would need to be adjusted and standardized to allow reproducible calibration of fluorescence intensity.

At this stage, CLE imaging can offer micron level resolution in discriminating ablated from nonablated liver tissue and may, therefore, be of interest as a novel imaging tool in liver ablation research. If clinical translation is considered in the future, technical limitations imposed by CLE imaging depth and probe vulnerability will have to be addressed.

The feasibility of utilizing needle based, diffuse optical spectroscopy to evaluate liver malignancy 32, 33 and steatosis 34 has been demonstrated in the past. A similar needle‐based approach could also be applied to increase the imaging depth of CLE, which is currently restricted to <200 μm. The smallest commercially available CLE probe has a diameter of 300 μm, which would enable insertion through the bore of a 24G needle which is smaller than liver core biopsy needles currently in routine use and, therefore, less likely to cause inadvertent injuries. Once placed within the bore of the needle, the CLE probe could be inserted into the liver parenchym under USS guidance. Correlation of USS and CLE images could then, respectively, provide anatomical and functional information about the adequacy of ablation therapy.

In the diagnosis and treatment of abdominal malignancy, there are a number of scenarios (e.g., transgastric fine needle aspiration of the pancreas, irreversible electroporation of the pancreas, liver biopsy) where a needle is intentionally inserted into malignant tissue. This is feasible because the risk of needle tract seeding is either small or outweighed by the clinical benefits of the procedure 35, 36, 37. To minimize the risk of needle tract seeding from needle‐based CLE, an ablation of the needle tract should be carried out which is advocated by some authors as routine practice in any laparoscopic liver ablation 3. In addition, if the procedure is being conducted anyway, the obtaining of CLE data through the same needle track simply adds to the benefits side of the risk evaluation and offers the possibility of increased data yield from the same clinical exercise. Further testing and validation in an experimental setting will be required to elucidate if this solution is feasible. Until feasibility has been confirmed, experimental CLE ablation monitoring should be restricted to superficial liver tumors that do not require a needle based approach.

Another technical issue is the CLE probe's vulnerability to high temperatures. The Cellvizio™ probes are only licensed to work within the range of normal body temperature whereas liver ablation may generate temperatures in excess of 100°C. This may prevent real time CLE monitoring within the center of an ablation zone but the temperature in the periphery of an ablated region is generally lower than in the center and rapidly deteriorates 38. Temperatures that are within the working range of a CLE probe can be expected within minutes of completing liver ablation therefore potentially allowing assessment during the same treatment session as near‐real time monitoring.

In summary, this article has assessed dual‐wavelength CLE for evaluating ablation therapy of focal liver lesions under laparoscopic guidance. Intraoperative CLE image acquisition during liver resection has been shown to be technically possible. In addition to a descriptive evaluation it was demonstrated that numerical analysis of fluorescence intensity could be employed to confirm the extent of liver ablation. Based on our findings, CLE may be of interest as a novel imaging modality in liver ablation research. Further investigation seems warranted to examine if technical limitations, that currently prevent a clinical translation of this approach, can be overcome.
